# Earliest Stone-Tipped Projectiles from the Ethiopian Rift Date to >279,000 Years Ago

**DOI:** 10.1371/journal.pone.0078092

**Published:** 2013-11-13

**Authors:** Yonatan Sahle, W. Karl Hutchings, David R. Braun, Judith C. Sealy, Leah E. Morgan, Agazi Negash, Balemwal Atnafu

**Affiliations:** 1 Department of Archaeology, University of Cape Town, Cape Town, South Africa; 2 Department of Sociology and Anthropology, Thompson Rivers University, Kamloops, Canada; 3 Department of Anthropology, George Washington University, Washington DC, United States of America; 4 Scottish Universities Environmental Research Centre, East Kilbride, United Kingdom; 5 Paleoanthropology and Paleoenvironment Program, Addis Ababa University, Addis Ababa, Ethiopia; 6 Department of Earth Sciences, Addis Ababa University, Addis Ababa, Ethiopia; University of Oxford, United Kingdom

## Abstract

Projectile weapons (i.e. those delivered from a distance) enhanced prehistoric hunting efficiency by enabling higher impact delivery and hunting of a broader range of animals while reducing confrontations with dangerous prey species. Projectiles therefore provided a significant advantage over thrusting spears. Composite projectile technologies are considered indicative of complex behavior and pivotal to the successful spread of *Homo sapiens*. Direct evidence for such projectiles is thus far unknown from >80,000 years ago. Data from velocity-dependent microfracture features, diagnostic damage patterns, and artifact shape reported here indicate that pointed stone artifacts from Ethiopia were used as projectile weapons (in the form of hafted javelin tips) as early as >279,000 years ago. In combination with the existing archaeological, fossil and genetic evidence, these data isolate eastern Africa as a source of modern cultures and biology.

## Introduction

A key component in prehistoric subsistence strategies, the invention of projectile weapons was a decisive advance over the thrusting spear [1]–[3]. The ability to wound or kill dangerous animals or enemies at a distance is considered one of the most significant adaptive advantages for Paleolithic hunters, reducing the likelihood of injury and increasing prey breadth [1]–[3]. In the Late Pleistocene, complex projectiles such as the bow-and-arrow probably contributed to the technological advantage enabling *Homo sapiens* to expand out of Africa and outcompete Neanderthals [3].

At Kathu Pan, in South Africa, Middle Pleistocene hominins made hafted stone-tipped hunting spears ∼500 thousand years ago (ka); these were, however, not projectiles but hand-delivered thrusting weapons [4]. In addition, the stratigraphic placement of the studied artefacts from Kathu Pan relative to the dated layers remains as yet controversial. Pointed wooden spears from Schöningen, Germany, dating to *∼*400 ka were likely used in hunting large game [5]. These were initially described as ranged weapons, but it has not been possible to definitively identify their mode of delivery [1], [2].

The identification of prehistoric projectile weaponry has been largely inferential. Paleolithic archaeologists suggest that mechanically-projected weapons, such as the bow-and-arrow, originated among modern humans in Africa *ca.* 100-50 ka [2], [3], [6], [7]. These inferences rely mostly on hafting mechanisms, morphometrics and weight thresholds of ethnographic specimens as a guide to identify prehistoric pointed artifacts suitable for use as projectile weapon tips. While such approaches have yielded useful insights, they are obviously constrained by the limitations of direct analogy with the ethnographic record. Micro- and macroscopic approaches, such as the analyses of hafting traces and macrofracture damage patterns, provide powerful independent means of inferring whether a pointed stone artifact was hafted and used [8], [9]. However, even these lines of evidence cannot confidently inform on the weapon delivery mechanism.

**Figure 1:**
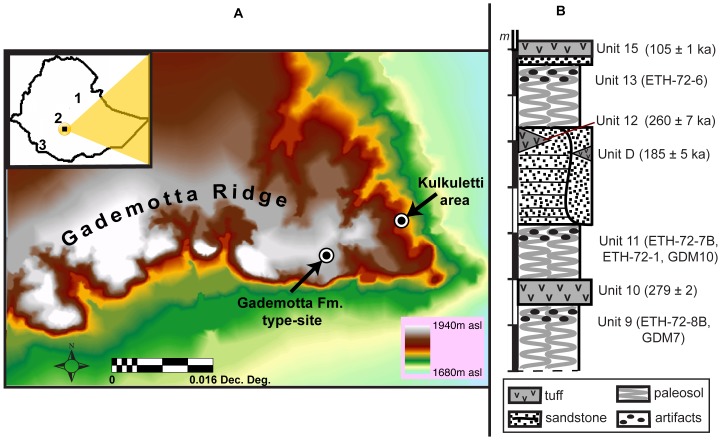
Location and stratigraphy of the Gademotta Fm. (**A**) A map showing the Gademotta ridge and major archaeological localities: the Gademotta type-site and the Kulkuletti area. Inset map shows the relative location of key later Middle Pleistocene sites in the Ethiopian rift, namely (1) Herto, (2) Gademotta, and (3) Omo Kibish. (**B**) A revised composite stratigraphic section of the Gademotta Fm. and the placement of major archaeological sites.

Here we report on multiple independent lines of evidence that strongly indicate that pointed obsidian artifacts recovered from sites in the Gademotta Formation (Fm.) of the Main Ethiopian Rift (Figure 1) represent composite projectile weapons that were incorporated into the hunting repertoire of *Homo* as early as >279 ka. Specifically, we apply the velocity-dependent microfracture approach (alongside the more commonly used ballistic methods of studying macrofracture damage patterns and Tip Cross-Sectional Area and -Perimeter) to Middle- and Late Pleistocene pointed artifacts to reliably identify the mode(s) of weapon delivery.

Microfracture features, such as fracture wings (one form of primary Wallner lines), are velocity-dependent ripple marks created when a crack force encounters intrinsic imperfections (such as bubble pores) in materials like obsidian [10], [11]. The analysis of these velocity-dependent fracture features provides a direct and objective tool for determining the velocity of fracture formation on stone artifacts. Specifically, it uses the geometry of the features along a crack plane of an impact-fractured stone artifact and the physical properties of the stone raw material to calculate the velocity of fracture formation [10], [11]. Details about the impact responsible for initiating the particular fracture, such as its type, can then be inferred from the velocity of the progressing fracture plane [11].

## Materials and Methods

The Gademotta Fm. archaeological site complex is located in the flanks of an ancient collapsed caldera in the central sector of the Main Ethiopian Rift (Figure 1A). It contains several hominin occupations spanning the Middle and Late Pleistocene [12]–[14]. The lowermost occupation horizon, sampled by sites ETH-72-8B and GDM7 in the type-site, has recently been redated by ^40^Ar/^39^Ar methods to >279±2 ka [12]–[14] (Figure 1B), using the most recent decay constants and age standards [15]. Sites ETH-72-7B, ETH-72-1, and GDM10 were previously bracketed between 279±2 and 185±5 ka [12]. New ^40^Ar/^39^Ar results reported here now provide a tighter minimum age constraint for these sites. The youngest occupation (ETH-72-6) is constrained to between 185±5 [12] and 105±1 ka [16] (Figure 1B).

^40^Ar/^39^Ar analyses were conducted on tephra samples collected from fine ash (Trench1-Step1) as well as pumice deposits (Trench1-Step2) of Unit 12 in the type-site (Figure 1B). Sample preparation and measurement for new ^40^Ar/^39^Ar analyses reported here largely followed the procedures and protocols for previous analyses from the same area [12], [13]. Decay constants and standard ages follow Renne *et al.* [15]. Values computed using Steiger and Jäger [17] and Renne *et al*. [15] are provided in Data S1. Uncertainties are provided at the 1σ level and include full analytical and systematic uncertainties; reported values are standard error of the mean (SEM), except where the mean square weighted deviation (MSWD)>1, where uncertainties are equal to 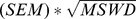. Ages published previously [12] have been revised here using decay constants and standard ages provided by Renne *et al*. [15] in order to maintain methodological consistency in the discussion of ages of sites. The revised age for the Aliyo Tuff in the Kibish Fm. was made using the published full Ar data in McDougall *et al*. [18] and the spreadsheet in Renne *et al.* [15].

Renewed research in the Gademotta Fm. was initiated in 2010. Archaeological sites were excavated using a grid-system of 1 m sq. Total stations (Leica TC307 and Leica Builder 505) were used to record the three-dimensional co-ordinates of each *in situ* artifact >20 mm. Detailed surveying and excavation methods are summarized elsewhere [13]. Previous [14] and renewed [13] excavations have recovered ∼44,000 stone artifacts from several Middle Stone Age (MSA) sites in the Gademotta Fm. *Débitage* classes and simple flakes dominate most of the assemblages [13], [14]. In particular, small *débitage* represents a major category (e.g. >44% of the total count of the ETH-72-8B assemblage [14]). This can be attributed to the brittle nature of obsidian [14], and on-site manufacturing and retouch activities. Retouched tools at Gademotta are abundant by MSA standards, with points and scrapers well represented in the formal tool categories. In total, 266 artifacts (223 from previous excavations; 43 from renewed ones) have been categorized under the pointed-tools category from sites ETH-72-8B, ETH-72-7B, ETH-72-1, ETH-72-6, GDM7, and GDM10 [13], [14]. This category consisted of Levallois points, typical and atypical Mousterian points, retouched points, points with basal thinning, bifacial points, and point fragments [13], [14].

The Gademotta artifacts are made almost exclusively on obsidian. Geochemical provenancing establishes that the obsidian used for artifact manufacture at Gademotta came from the nearby (<2.5 km distant) obsidian source at Kulkuletti/Worja [19], [20] (Figure 1A).

All 226 convergent artifacts (171 pointed tools and 95 point fragments) were examined; 141 were found to bear fracture patterns potentially attributable to impact from their use as weapons. All of these pieces are made on obsidian. Microfracture analysis assessed velocity-dependent fracture features to identify use-related precursory loading [11]. Edge-damage analysis documented the pattern and location of macroscopic fractures to differentiate impacts from projectile use [21]–[23]. Morphometric analyses of TCSA and TCSP were employed to assess the suitability of pointed pieces for hafting and use as projectile tips, compared with ethnographic, archaeological and experimental specimens [2], [3], [24], [25]. The combined result of these independent analyses has enabled a confident identification of the weapon delivery mechanisms of at least some of the Gademotta pointed artifacts [13].

Fracture velocity analyses of pointed artifacts identified and measured plane fracture wings. Fracture wings (FWs) are V-shaped, with their apex pointing toward the direction of fracture propagation [10], [11], [26]. The angle of divergence of plane FWs on a given crack front allows the calculation of fracture velocity for an artifact of a raw material of a known distortional wave velocity. The higher the velocity of impact the narrower the angle of divergence of a fracture wing [11].

Fracture velocity analysis involved multiple stages: i) determination of the physical properties of the obsidian raw material exploited by prehistoric inhabitants of the Gademotta sites, as described below; ii) microscopic investigation of fracture surfaces on pointed pieces; iii) capturing of microfracture features in photomicrographs; iv) measurement of dimensions of microfracture features; v) calculation of instantaneous fracture velocity.

Non-artifactual obsidian samples (*n* = 32) were collected from the Kulkuletti/Worja primary source [19], [20] and cut into 5 cm cubes using a lapidary saw. Young’s Modulus (*E*) and Poisson’s Ratio (*v*) for the Kulkuletti/Worja obsidian were determined by the pulse method using the NDT James Instruments MK IV ultrasonic transducer in the National Museum of Ethiopia, Addis Ababa. These properties were used to calculate the Modulus of Rigidity (*G*). The distortional wave velocity (*C_2_*) of the Worja obsidian was computed from *G* and density (*ρ*) of the Kulkuletti/Worja obsidian. Density was measured in the Concrete Materials and Structural Integrity Research Unit (CoMSIRU) of the Department of Civil Engineering, University of Cape Town. The following formulae and procedures detailed in Hutchings [11] were used in the calculation of the physical properties of the Worja obsidian:
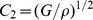


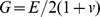



**Figure 2:**
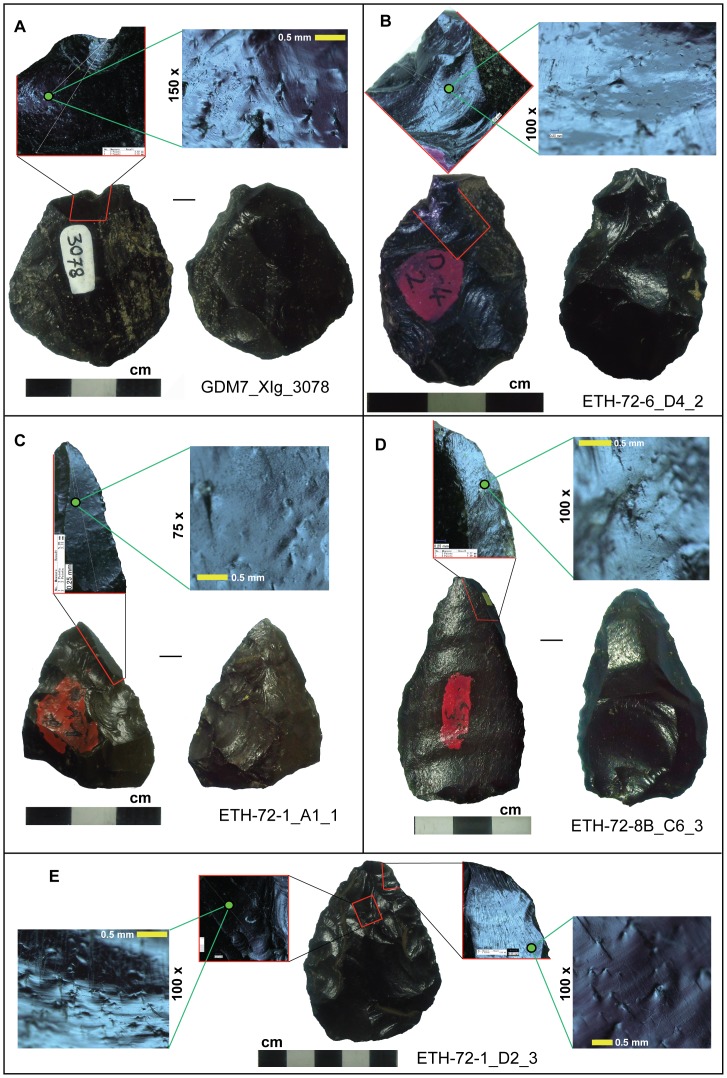
A sample of Gademotta pointed artifacts exhibiting micro- and macrofracture features indicative of projectile weaponry. (**A**, **B**) fracture wings on transverse fractures; (**C**, **D**) fracture wings on burin-like fractures; (**E**) impact fractures on two fracture fronts on the distal portion.

**Figure 3:**
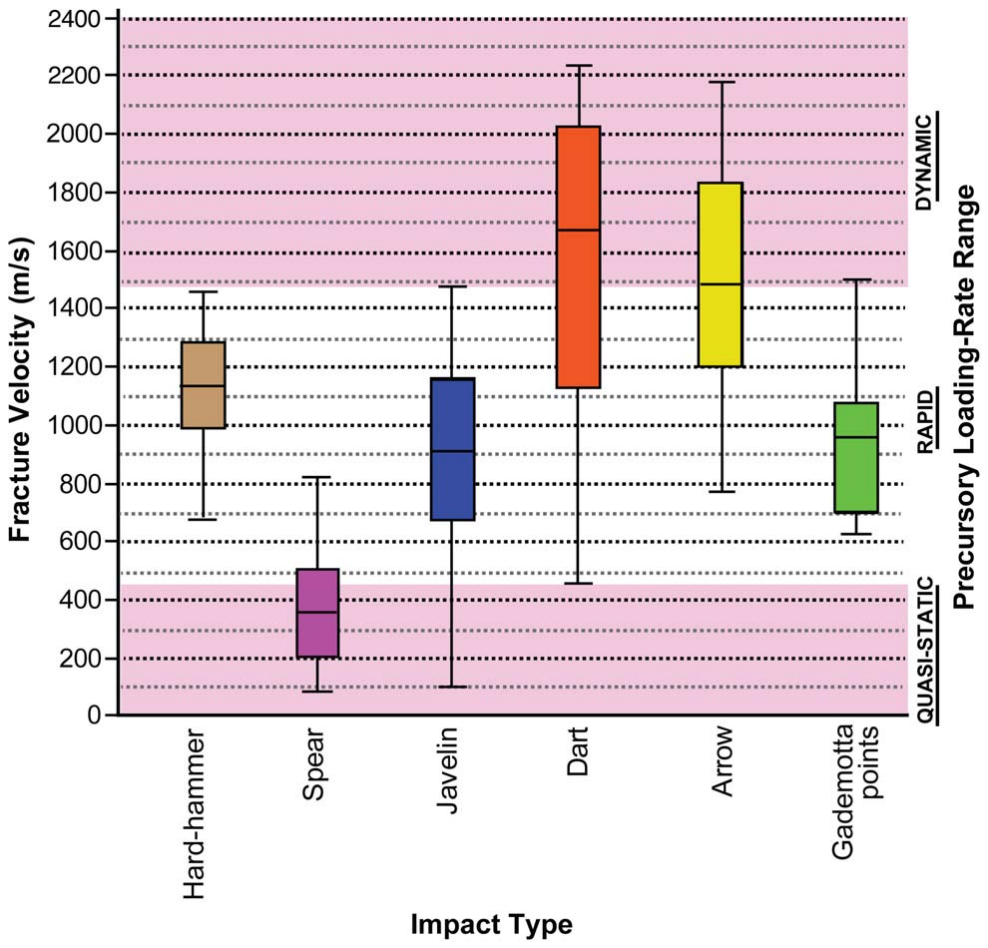
Box-and-Whisker plots of instantaneous fracture velocities for various impact types. Comparison established by experimental work using obsidian raw material with a distortional wave velocity (*C*_2_) of 3865 m/s [11]. Boxes represent inter-quartile ranges; the horizontal lines inside the boxes represent the median values; the tails represent the non-outlier range.

Instantaneous fracture velocity (*C*) was calculated from the angles of FWs (Figure 2) using the equation *C* = cos ([*ψ*/2]* *C*_2_); where *ψ* is the angle of divergence of a plane fracture wing. Measurements of angle of divergence were conducted on digital versions of photomicrographs using the built-in package on the Keyence VHX-600 (3CCD) digital microscope housed in the NME, and independently using the external software packages MB Ruler 4.0 (http://markus-bader.de/MB-Ruler) and ImageJ 1.440 (http://imagej.nih.gov/ij). Table S1 summarizes material properties of the Worja/Kulkuetti obsidian. The computed instantaneous fracture velocity values for the Gademotta pointed pieces are provided in Table 1. Results were interpreted using instantaneous fracture velocity values and precursory loadings experimentally established using obsidian from a North American source with material properties analytically identical to the Kulkuletti/Worja obsidian [11] (Figure 3).

**Table 1:** GDM points with fracture velocity values beyond the range experimentally documented for thrusting spears [11] as well as macrofracture patterns considered DIFs [21]–[23].

Specimen ID	*ψ* (°)	*C* (*m/s*)	Damage details
ETH-72-1_D2_3	155	865	Tip; burin-like fracture with step termination; FL = 16 mm
ETH-72-8B_A3_5	153	933	Tip; burin-like fracture with step termination extending from tip to end of the right mediolateral edge. FL = 17 mm.
ETH-72-6_D4_2	153	933	Tip; transverse fracture; step-terminating bending fracture snaps off tip; FL = 9 mm.
ETH-72-8B_A3	152	966	Tip; transverse fracture with step termination; FL = 16 mm; very tip snapped
ETH-72-8B_D4_4	150	1034	Tip; burin-like fracture; FL = 10.2 mm
ETH-72-8B_C15_17	150	1034	Base; transverse fracture snapping piece; FL = 33 mm; no major distal fracture
ETH-72-8B_A3_4	149, 142	1068, 1301	i) Base; transverse fracture; FWs on snapped surface; ii) Tip; step-terminating burin-like fracture extending from tip to the right distolateral edge; FL = 9.5 mm
GDM7_IXg_3078	143	1268	Tip; transverse fracture removing entire tip of piece; FL = 7 mm
ETH-72-1_A1_1	139	1399	Tip; burin-like fracture extending from tip to the distolateral portion; proximal end is snapped; FL = 14.5 mm
ETH-72-8B_C6_3	136	1497	Tip; burin-like fracture on the right ventral side; FL = 30 mm. Another step-terminating fracture on the proximal tip of the dorsal side; FL = 8.5 mm

*ψ = *angle of divergence of FWs; *C = *instantaneous fracture velocity; FWs = fracture wings; FL = fracture length.

The identification of artifact edge damage was largely conducted at a macroscopic scale. A hand lens and low-power (<20x) magnification on binocular reflected light microscope were employed infrequently. Macroscopic fracture types most commonly considered diagnostic of impact from use of pointed pieces as weapon tips include: i) burin-like fractures; ii) flute-like fractures; iii) transverse fractures with terminations other than snaps that were inflicted after the artifact was retouched; iv) bifacial spin-off fractures; v) unifacial spin-off fractures with a fracture length of >6 mm [21]–[23]. Bending fractures with step terminations have often been presented as an additional, distinct type of fracture diagnostic of projectile impact. However, impact fracture initiations are commonly bending, rather than cone [27]. In order to avoid confusion, bending fractures are treated here as part of *transverse fractures* [22]. Fractures that retain negative bulbs and those with feather terminations are often indicative of a manufacturing process, rather than impact damage, as these are most likely percussion induced [27] (but see [28]). As a result, they required a more careful examination (for instance, the order of occurrence of fractures) [22], [23]. Impact fractures are not exclusively limited to the distal tips and distolateral portions of pointed pieces. Less frequently, they can occur along the medial/proximal portion of a pointed piece where the impact snaps a piece within a haft [29]. Additional lines of evidence suggestive of hafted weapon use include treatment of the proximal end of pointed pieces to facilitate hafting, and ventral flaking of the distal tip [8], [30].

Artifact morphology is one important variable that prehistoric hunters optimized to achieve the desired aerodynamic qualities and penetrative abilities of hafted points [2], [3], [25]. A recent work [31] shows that the morphometric approach of TCSA/P is not applicable to all assemblages of points. We recognize that no approach to identifying prehistoric weaponry will be successful in all cases [13]. We have used the TCSA/P method in combination with micro- and macrofracture approaches, including evidence for hafting (in the form of microwear data and modification on the proximal end of points) and ventral flaking. More importantly, it has to be noted that the TCSA/P approach can only serve as a method for assessing the *potential* of pointed artifacts to have served as tips of projectile weapons [24]. A more confident identification of projectile weaponry requires data from other independent approaches.

TCSA and TCSP were calculated following the methods detailed by Shea [2], and Sisk and Shea [24]:
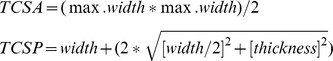



For TCSP, values from the more restrictive measure of triangular, rather than rhomboidal, cross-section were used, as recommended by Sisk and Shea [24]. Dimensional measurements for morphometric analyses were collected on 113 of the 141 Gademotta pointed pieces that showed impact fractures. These pieces came from all six of the Gademotta sites studied [13], [14] (Figure 1B). The remaining 28 pieces were unsuitable for this analysis because the measurements necessary for the calculations of TCSA and TCSP could not reliably be identified on them. TCSA and TCSP values were statistically compared with experimental [25] and substantially younger archaeological [32] samples. The Brunner-and-Munzel-generalized-Wilcoxon (BMgenW) Test has been employed as it provides a powerful pairwise comparison of sets of data where variances are not equal and distributions are not symmetric.

## Results

^40^Ar/^39^Ar analysis on samples Trench1step1 and Trench2step2 yielded analytically indistinguishable results, with a combined isochron age of 260±7 ka (Figure S1, Data S1). The stratigraphy of the Gademotta Fm. has been divided into several interstratified paleosol and volcanic ash units [12]–[14]. The newly established age for the upper portion of Unit 12 provides a more definitive [12] minimum age for occupations in the underlying Unit 11 paleosol (Figure 1B).

Eighteen impact-induced microscopic fracture features were identified on 16 of the 141 pointed artifacts studied (Figure 2A–E). The ages of these pieces ranged from ∼105 ka to >279 ka. Half of these pieces with fracture velocity data (i.e. 8 out of 16) derive from the two oldest sites in the Formation (namely ETH-72-8B and GDM7) dating to >279 ka (Figure 1B). All of these features are plane fracture wings [11], [26] (Figure S2). Computation yielded fracture velocity estimates ranging from 625 to 1495 m/s (Table 1 and Figure 3). Previous experimental work has shown that impact fracture velocities associated with thrusting spears attain a maximum of 813.5 m/s; for javelins (i.e. throwing spears) they can reach 1472.8 m/s [11] (Figure 3). About 73% (12 out of 16) of the pointed artifacts from Gademotta exhibit fracture velocity values beyond the range experimentally established for thrusting spears (Figure 3 and Table 1). Analyses revealed that the direction of fracture propagation, inferred from the orientation of fracture wings (Figure 2 and Figure S2), is from the tip on 14 of the 16 artifacts (on the two remaining pieces the fracture fronts of interest are on snapped basal surfaces). These pointed artifacts were, hence, used in a longitudinal fashion (i.e. for stabbing, thrusting, and/or distance-penetrating) [10], [11], [26]. The fractures containing FWs on these pieces were inflicted after the shaping/resharpening removals. This provides further evidence that the microfracture features under investigation were the result of use-related rather than manufacturing impact [22], [23]. Only fracture velocities for arrows and darts extend into the *dynamic* precursory loading regime [11] (Figure 3). Only one Gademotta point (Figure 2D), exhibits a value within this range, at 1497 m/s (Table 1). However, despite its high fracture velocity value, this specimen bears a morphometric affinity (TCSA = 162.37; TCSP = 70.91) to spears rather than arrows/darts [24], [25]. We infer that it was the tip of a javelin [11] (Figure 3).

Macroscopic edge damage analysis shows that just over 81% (13 out of 16) of the pointed pieces with impact-induced microfractures also display macrofeatures referred to as diagnostic impact fractures (DIFs) [21]–[23]. Impacts are limited to the distal (i.e. pointed) tips of all but 3 of the 16 artifacts (Table 1). Damage on the medial-basal portions of the remaining 3 artifacts may have resulted when the pieces yielded to stress from impact while in a haft [29]. DIFs range from burin-like (31.2%; 5 artifacts) to transverse fractures with step termination (37.5%; 6 artifacts) [21], [22] (Figure 2). Proximal thinning is positively documented on 2 of the16 pieces with impact-induced microfractures, in addition to 12 others from the overall assemblage from multiple sites (*n* = 141). This evidence for artifact modifications to facilitate hafting supports previous inferences from use-wear analyses in which microscopic striations on the surfaces of Gademotta points were interpreted as the result of hafting [33]. Two (12.5%) pieces exhibit unifacial spin-off fractures <6 mm in length which are not considered DIFs [21], [22].

**Figure 4:**
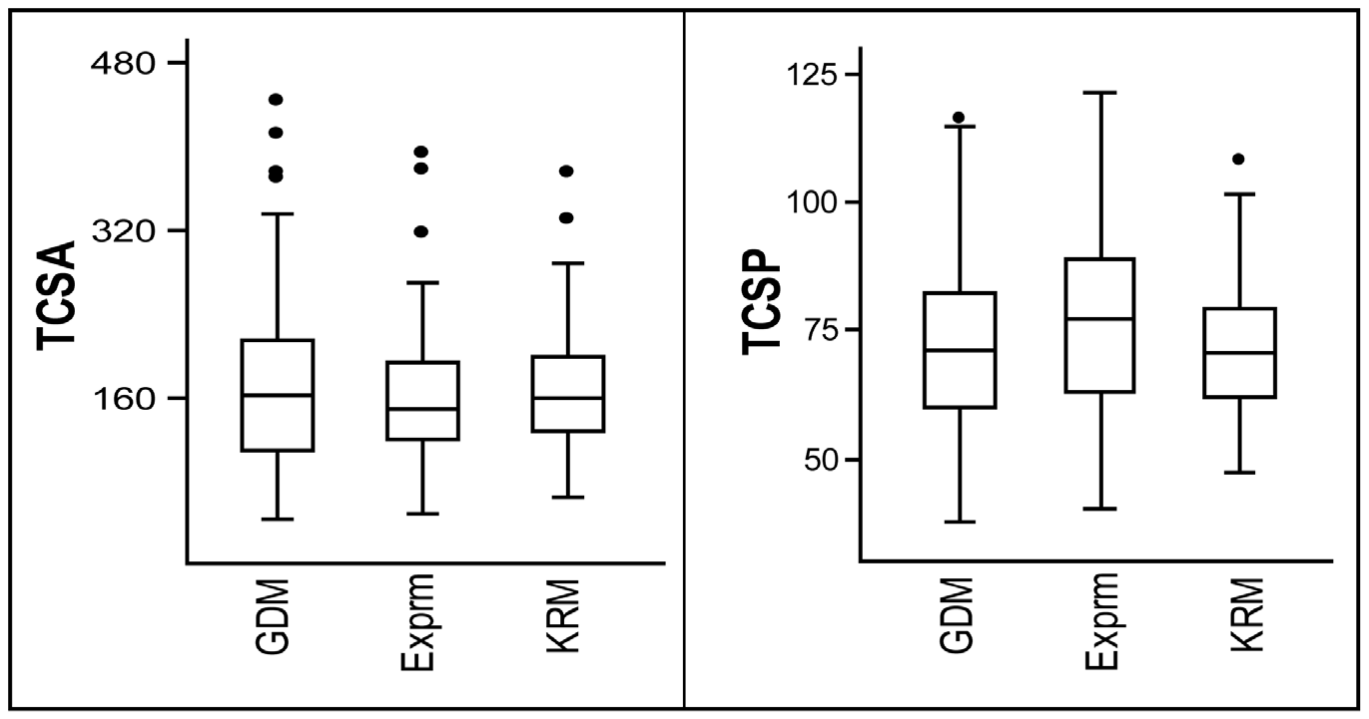
Box-and-Whisker plots of TCSA and TCSP comparisons. TCSA and TCSP plots of pointed pieces from experimental spear tips [25] (Exprm); Klasies River main site MSA I [32] (KRM); Gademotta (GDM). Solid dots represent outlier values.

Morphometric analyses show that both the TCSA and TCSP of the GDM point assemblage (*n* = 113 from all six sites in the Formation) are statistically indistinguishable (BMgenW Test −0.3242; *p* = 0.7475 for TCSA; 1.0672; *p* = 0.293 for TCSP; Figure 4) from an experimental spear point assemblage made to replicate Middle Paleolithic Levantine pointed artifacts (*n* = 28) [25]. Based on morphological parameters, these experimental points are described as effective hunting spears [25]. The GDM points are also not significantly different (BMgenW Test 0.4409; *p* = 0.6598 for TCSA; −0.1211; *p* = 0.9037 for TCSP; Figure 4) from an archaeological assemblage of pointed pieces (*n* = 71) from a substantially younger (90 ka) MSA context at Klasies River main site (KRM), South Africa [32]. Associated fauna from the MSA at KRM attests to the hunting prowess of these hominin populations [34].

Combined results from the independent approaches presented here show that certain Gademotta points were used as javelin tips from as early as >279 ka through to ∼105 ka.

## Discussion

The evidence for the earliest composite projectile weaponry at Gademotta >279 ka is significant because it provides direct evidence for a highly advantageous, complex technology that pre-dates the emergence of *H. sapiens* [35]. Complex behaviors are therefore found amongst more than one hominin species, and are not unique to *H. sapiens* [36]–[38]. The evolution of our lineage was shaped by complex interactions between biology, environment and culture [39]. Throwing of composite stone-tipped javelins was one stage in a long process with much deeper evolutionary roots. Roach and colleagues [40] have recently shown that hominins’ ability to throw effectively depends upon a cluster of features in the anatomy of the shoulder, and that this first appeared in *H. erectus* about 2 million years ago. They argue that throwing – leading to an increase in hunting success – helped to shape the evolutionary trajectory of *Homo*.

Prominent models for modern human origins propose that alterations in the costs and benefits of adaptation, triggered by paleoenvironmental changes that created stable ecological settings, contributed to the emergence of new adaptive strategies between 350 and 250 ka among populations ancestral to *H. sapiens* [41], [42]. The appearance and maintenance of these strategies reflects an overall pattern of increased reliance on social and technological information [43], [44]. This may have provided the evolutionary advantages that later allowed our species to expand to every known terrestrial habitat and outcompete other hominin species [3]. There is indirect/circumstantial evidence for the presence of projectile technologies prior to 200 ka [5], [8], [45], [46], but conclusive evidence has not, thus far, come from the period predating ∼80 ka. Our study shows that it was present by at least 280 ka. We suggest that the manufacture and use of multi-component stone-tipped throwing spears not only conferred significant advantages during the hunt, but also demonstrates complex behaviors not previously securely associated with this period.

In recent years, studies of the behavior of Middle Pleistocene hominins, including early *H. sapiens*, have moved away from a list of traits considered ‘behaviorally modern’ to focus instead on evidence of complex behaviors [37], [47]. The onset of complex technological adaptations is probably linked to a series of factors that allow for more efficient processing of information and extraction of resources from a broader niche [39], [44], rather than sudden neural alterations among *H. sapiens* [38]. Among such adaptations are advances in hunting technologies including hafting and projectile weapon use [3], [9], [37], [43], [48]. The making of retouched pointed pieces, their hafting to a shaft and use as projectile hunting weapons is among innovations that have cognitive implications, even in the absence of direct evidence for transformative technologies, such as the use of irreversibly-altered adhesives [37], [49]. Sites in the Gademotta Fm. represent the earliest instance of a clearly MSA occupation from a securely dated context [12], [13]. Even the oldest (>279 ka) sites of ETH-72-8B and GDM7 in this Formation contain retouched tools (including unifacial and bifacial points) that compare in their technological variability with assemblages from younger sites [12], [13], [50]. The present direct evidence for the earliest stone-tipped projectile weapons is consistent with the relatively early occurrences of retouched points [13], [14] and hafting [33] at these sites.

The late Middle- and earlier Late Pleistocene archaeological record from northeastern Africa particularly emphasizes the greater antiquity of behaviors considered complex [35], [37]. This evidence has not, however, been prominent in discussions of this topic; to date, early complex behaviors are most extensively documented from other regions of the continent and later time periods, e.g. [7], [9], [37], [43], [51], [52]. Earlier studies [14], [33] have suggested that the Middle Pleistocene record of the Gademotta Fm. attests to unique technological stability across much of the length of the MSA. This may be attributable to the presence of a nearby obsidian source, and the location of the site-complex in a near-lake and ecotonal environment [53]. These would have supported successful and continuous habitation in the region and created demographic and environmental settings conducive to the emergence of such innovative technologies [44].

Currently, fossil evidence for the world’s earliest *H. sapiens* derives from sites in the Ethiopian rift, namely Omo Kibish and Herto [15], [54], [55] (Figure 1A). At Herto (Figure 1A), all three early *H. sapiens* crania were carefully de-fleshed. These specimens were not associated with post-cranial skeletal remains; this is unlikely to be due to preservation, since conditions were good enough to preserve the relatively delicate skull of a juvenile individual [55]. This find therefore provides strong indications of ritual practice through post-mortem manipulation and curation of human remains by 154 ka [54], [55]. Recent geochemical provenancing supports the exploitation of obsidian raw material by these early modern human populations from as far as 289 km away, in addition to several nearby sources [56]. The Herto early humans therefore had a working knowledge of such distant resources on the ancient landscape and/or embraced complex social practices involving trade/exchange/gift-giving. The behavioral capacity of Middle Pleistocene hominin populations from the Kibish Fm. of the Ethiopian Omo basin (Figure 1A) is inferred to have been essentially similar to Late Pleistocene inhabitants of the same region [47], [50]. Several additional lines of evidence substantiate that the broader region supported stable adaptations across a long period of time. By 125 ka, humans in this region had occupied coastal areas of the Eritrean Red Sea and were probably exploiting marine resources [57]. Population expansion and/or contact across the early Late Pleistocene is evidenced in the wider region from the presence of the region-specific Nubian techno-complex at Gademotta by >105 ka [14], as well as elsewhere in the Ethiopian and Eritrean rifts (Kone [58]; Aduma [59]; Asfet [60]) and across the Red Sea in southern Arabia by *∼*106 ka [61].

Current notions that Middle Pleistocene hominins were behaviorally dramatically different from their Late Pleistocene descendants [38] seem to be an artifact of our patchy knowledge of the archaeological record and data from disparate fields of study [39], [47], [62]. Accumulating genetic, fossil, archaeological and other lines of evidence from eastern Africa, especially the fact that modern humans who successfully spread beyond Africa all descend from the L3 genetic lineage prominent in eastern (but not southern) Africa, [15], [55]–[57], [63] point to the region as a significant source for modern humans.

## Supporting Information

Figure S1 **Graphs of (A) relative probability and (B) inverse isochron of single crystal total fusion analyses for sanidines for samples T1S1, T1S2 (in red), and combined results from both samples.** Xenocrysts are shown in pink on **A**, and are excluded from age calculations; they are not included on **B**.
(TIF)

Figure S2 **Pictures showing (A) a fracture surface containing FWs sampled for analysis from a locus at 34.7% of the crack length; (B) a photomicrograph of plane FWs; and (C) the measurement of angle of divergence of a prominent FW.**
(TIF)

Table S1 **Material properties of the Kulkuletti/Worja obsidian.** Data were collected via the pulse method. The ultrasonic transducer was set to 1 pulse per second for all measurements. The average *E* value was converted to Newton/m^2^, yielding a value of 8.9425e+10 N/m^2^, cf. [11]. Density of the Kulkuletti/Worja obsidian was determined via the immersion method and is 2.394 g/cm^3^.
(PDF)

Data S1 Full Ar data for Trench1step1 and Trench1step2 samples.
(XLS)
