# Correction: Earliest Stone-Tipped Projectiles from the Ethiopian Rift Date to >279,000 Years Ago

**DOI:** 10.1371/journal.pone.0126064

**Published:** 2015-04-29

**Authors:** Yonatan Sahle, W. Karl Hutchings, David R. Braun, Judith C. Sealy, Leah E. Morgan, Agazi Negash, Balemwal Atnafu

In the Materials and Methods section, there is an error in the TCSA equation. "max. width" is incorrectly repeated in the formula for TCSA, and should be "max. thickness" the second time. Please view the complete, correction equation here:

TCSA=(max.width*max.thickness)/2
