# Supplementary figures and images for: Earliest Stone-Tipped Projectiles from the Ethiopian Rift Date to >279,000 Years Ago
Source: PLoS One. 2013 Nov 13;8(11):e78092. doi: 10.1371/journal.pone.0078092 (PMC3827237; doi:10.1371/journal.pone.0078092)

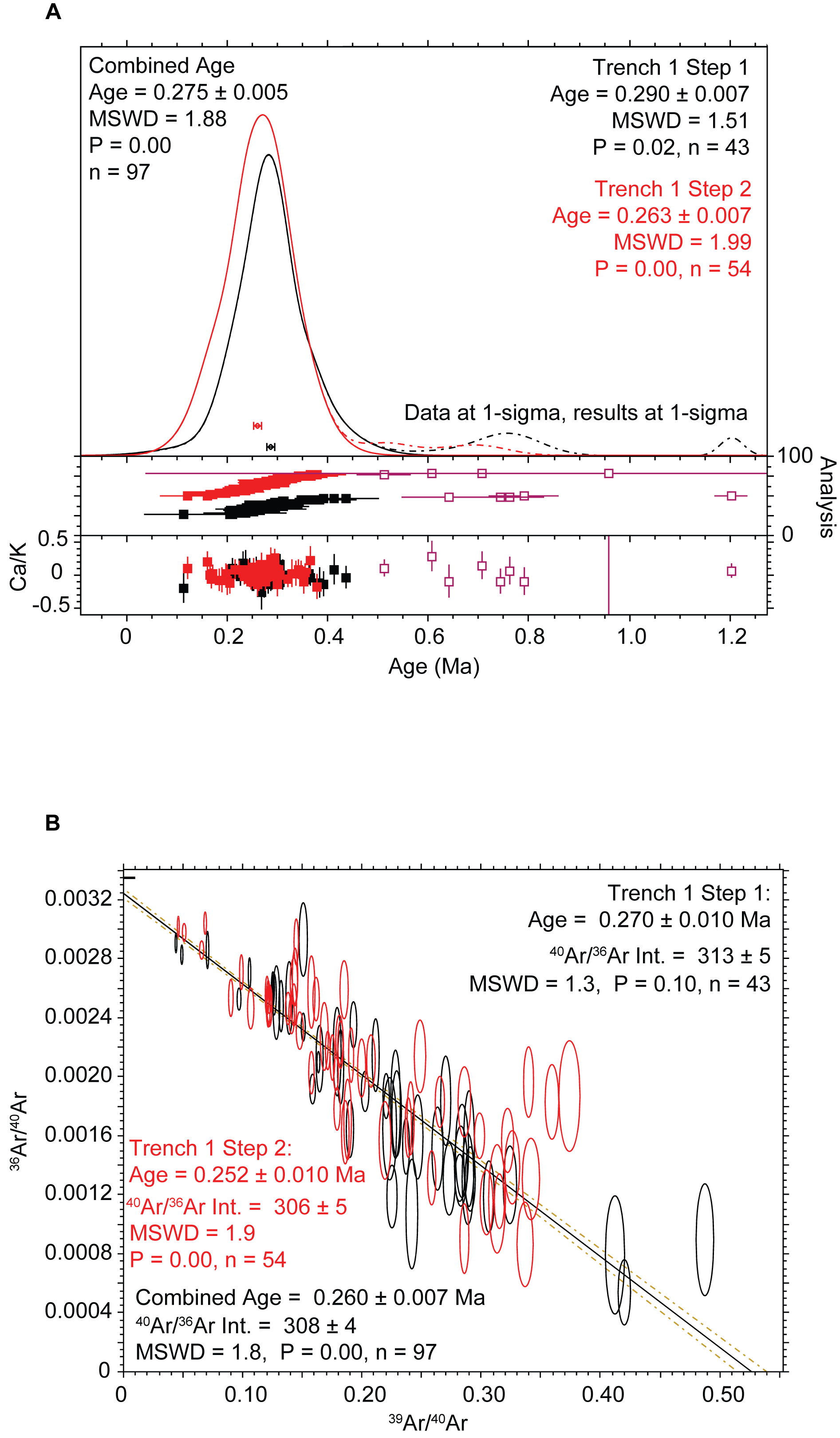

Supplement: Figure S1 — Graphs of (A) relative probability and (B) inverse isochron of single crystal total fusion analyses for sanidines for samples T1S1, T1S2 (in red), and combined results from both samples. Xenocrysts are shown in pink on A, and are excluded from age calculations; they are not included on B. (TIF) [file pone.0078092.s001.tif]

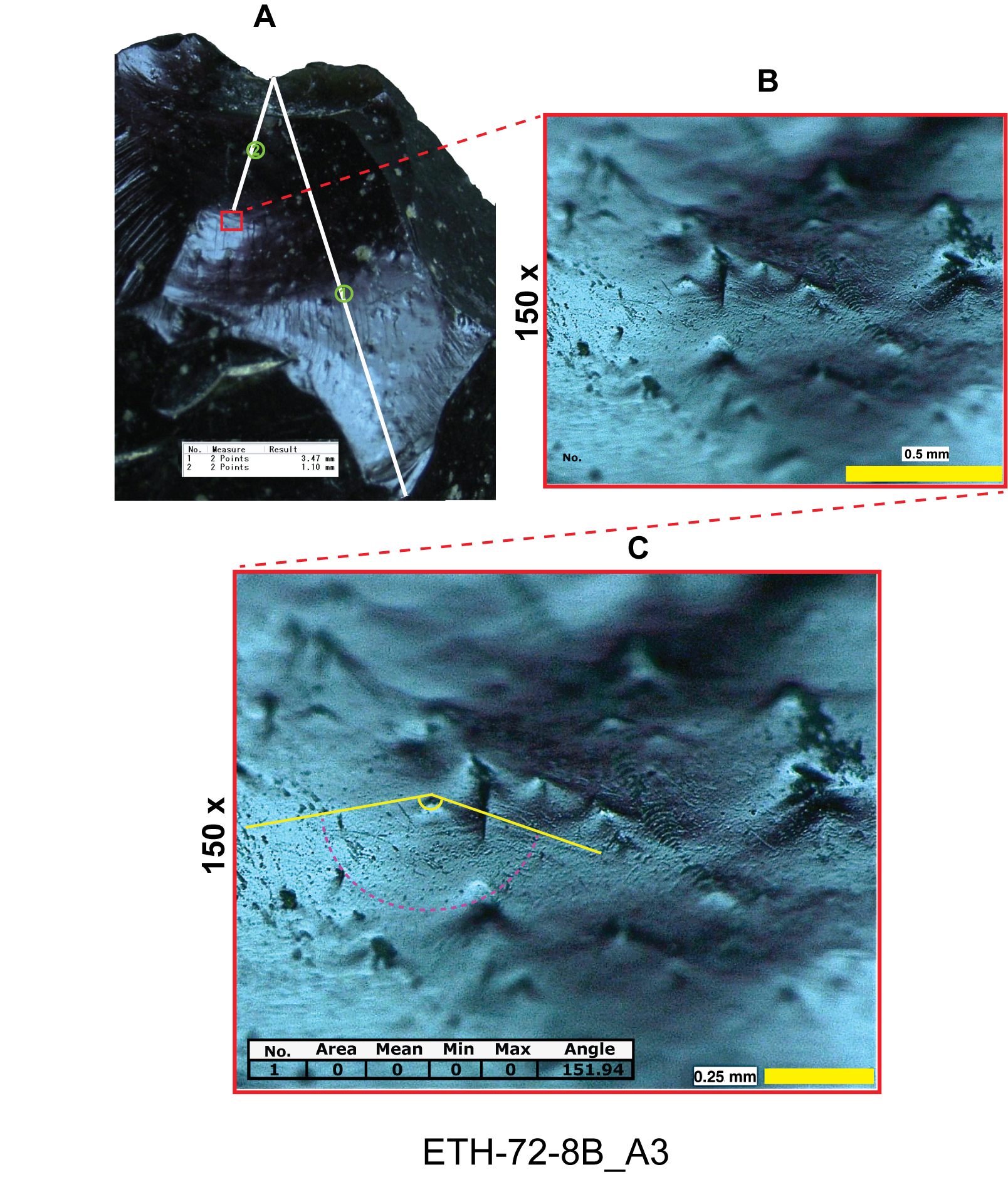

Supplement: Figure S2 — Pictures showing (A) a fracture surface containing FWs sampled for analysis from a locus at 34.7% of the crack length; (B) a photomicrograph of plane FWs; and (C) the measurement of angle of divergence of a prominent FW. (TIF) [file pone.0078092.s002.tif]
