# Supplementary material for: Earliest Stone-Tipped Projectiles from the Ethiopian Rift Date to >279,000 Years Ago
Source: PLoS One. 2013 Nov 13;8(11):e78092. doi: 10.1371/journal.pone.0078092 (PMC3827237; doi:10.1371/journal.pone.0078092)
Supplement: Table S1 — Material properties of the Kulkuletti/Worja obsidian. Data were collected via the pulse method. The ultrasonic transducer was set to 1 pulse per second for all measurements. The average E value was converted to Newton/m2, yielding a value of 8.9425e+10 N/m2, cf. [11]. Density of the Kulkuletti/Worja obsidian was determined via the immersion method and is 2.394 g/cm3. (PDF) [file pone.0078092.s003.pdf]

Table S1. Material properties of the Kulkuletti/Worja obsidian. Data were collected via the pulse method. The ultrasonic transducer was set to 1 pulse per second for all measurements. The average *E* value was changed to Newton/m^2, yielding a value of 8.9425e+10 N/m^2 [*cf. 18*: table 1]. Density of the Kulkuletti/Worja obsidian was determined via the immersion method and is 2.394 g/cm^3.

Sample ID	P-Distance (cm)	Young's Modulus (*E*)	Poisson's Ratio (*v*)	Transit Time	Voltage (high/low)
KUL 1-1	5.4	12.92e+6 psi	0.17	11.2 μ sec	H
KUL 1-2	5.1	12.98e+6 psi	0.17	11.1 μ sec	H
KUL 1-3	5.4	12.76e+6 psi	0.17	11.9μ sec	H
KUL 1-4	5	13.32e+6 psi	0.17	11.4 μ sec	H
KUL 1-5	6.4	12.92e+6 psi	0.17	15.7 μ sec	H
KUL 2-1	6.2	12.99e+6 psi	0.17	11.7 μ sec	H
KUL 2-2	6.3	12.82e+6 psi	0.17	13.1 μ sec	H
KUL 2-3	6	13.2e+6 psi	0.17	11.3 μ sec	H
KUL 2-4	5.4	12.9e+6 psi	0.17	13.8 μ sec	H
KUL 2-5	5.5	12.89e+6 psi	0.17	10.7 μ sec	H
KUL 3-1	6.4	12.94e+6 psi	0.17	11.8 μ sec	H
KUL 3-2	6	12.74e+6 psi	0.17	12.1 μ sec	H
KUL 3-3	5.76	12.88e+6 psi	0.17	11.1 μ sec	H
KUL 3-4	5.8	12.91e+6 psi	0.17	11.1 μ sec	H
KUL 3-5	6.42	12.88e+6 psi	0.17	14.9 μ sec	H
KUL 4-1	5.5	12.92e+6 psi	0.17	11.3 μ sec	H
KUL 4-2	5.2	13.0e+6 psi	0.17	11 μ sec	H
KUL 4-3	5.96	13.1e+6 psi	0.17	11 μ sec	H
KUL 4-4	5.72	12.98e+6 psi	0.17	13.1 μ sec	H
KUL 4-5	5.5	13.13e+6 psi	0.17	11.4 μ sec	H
KUL 5-1	5.9	12.93e+6 psi	0.17	13 μ sec	H
KUL 5-2	4.89	13.12e+6 psi	0.17	11.1 μ sec	L
KUL 5-3	5.83	12.97e+6 psi	0.17	11.6 μ sec	H
KUL 5-4	5.97	12.94e+6 psi	0.17	12.2 μ sec	H
KUL 5-5	4.9	13.1e+6 psi	0.17	1.6 μ sec	L
KUL 6-1	5.58	13.34e+6 psi	0.17	12.3 μ sec	H
KUL 6-2	5.49	12.96e+6 psi	0.17	11.7 μ sec	H
KUL 6-3	5.45	12.98e+6 psi	0.17	10.3 μ sec	H
KUL 6-4	5.2	12.99e+6 psi	0.17	11.8 μ sec	H
KUL 6-5	5.16	12.66e+6 psi	0.17	12.8 μ sec	H
KUL Test1	5.0	12.91e+6 psi	0.17	11.3 μ sec	H
KUL Test2	5.0	12.96e+6 psi	0.17	12.1 μ sec	H
AVERAGE		***12.97 psi***	***0.17***		
